# Synergistic antitumor effect of resveratrol and sorafenib on hepatocellular carcinoma through PKA/AMPK/eEF2K pathway

**DOI:** 10.29219/fnr.v65.3602

**Published:** 2021-10-13

**Authors:** Meili Gao, Chun Deng, Fan Dang

**Affiliations:** Department of Biological Science and Engineering, The Key Laboratory of Biomedical Information Engineering of Ministry of Education, School of Life Science and Technology, Xi’an Jiaotong University, Xi’an, China

**Keywords:** hepatocellular carcinoma, resveratrol, sorafenib, PKA/AMPK/eEF2K pathway

## Abstract

Although sorafenib (Sor) is the only effective drug for hepatocellular carcinoma (HCC), its therapeutic potential to date is mainly limited to the low tumor response. This study was designed to explore whether resveratrol (Res) could potentiate the anticancerous activity of Sor. We used HepG2 and Huh7 HCC cell lines and BALB/c nude mice for *in vitro* and *in vivo* studies, respectively. The cultured cell lines and tumor induction in the mice were treated with different concentrations of Res and Sor alone, and the combination of Res and Sor to observe the antitumor effects. Significant inhibitory effects were observed in the combined treatment of Res and Sor compared to Res and Sor alone treatments both *in vitro* and *in vivo* as demonstrated by significantly high number of S phase cells and apoptotic cells. Moreover, these findings were accompanied by the reduction of CDK2, CDC25A, PKA, p-AMPK, and eEF2K protein levels and the increment of cyclin A, cleavage caspase-3, caspase-8, and caspase-9 protein levels. The combinational treatment exhibited more significant anticancerous effect than the Res and Sor alone treatments in mice-bearing HepG2 xenograft. Overall, our results suggest that PKA/AMPK/eEF2K pathway is involved in the synergistic anticancerous activity of Res and Sor combination treatment in HCC cells. Thus, Res and Sor combination therapy may be promising in increasing the tumor response of Sor in the future.

## Popular scientific summary

- Resveratrol potentiates the inhibited proliferation of sorafenib in hepatocellular carcinoma cells.
- Resveratrol and sorafenib combination treatment synergistically increased cells in S phase and apoptotic cells.
- Combination of resveratrol and sorafenib have synergetic effect in reducing the PKA/AMPK/eEF2K pathway and inhibiting the tumor *in vivo*.

As one of the most common malignancies, hepatocellular carcinoma (HCC) is the second leading cause of cancer-related deaths worldwide (1). It represents the most common histological subtype and accounts for 90% liver cancers (2, 3). HCC is predominant in Asian countries, including China, Mongolia, Southeast Asia, Sub-Saharan Western, and Eastern Africa (4). In China, due to the high prevalence of HBV, HCC is the second most common cause of cancer-related mortality with an estimated 598,000 deaths annually (5, 6).

Using screening programs for earlier diagnosis has shown that most HCC patients are at the intermediate- or advanced-stage of the disease (2, 5, 6). For these HCC patients, transcatheter arterial chemoembolization (TACE) and the multi-kinase inhibitor of sorafenib (Sor) are the two approved therapies (7). As a novel diaryl urea compound, Sor has shown the antiproliferative effects in HCC cell lines. It also decreased the tumor angiogenesis and tumor-cell signaling, while increased the apoptosis in a mouse model (2). Sor is the only approved systemic therapy to improve overall survival (OS) in patients with advanced HCC (1, 5, 8, 9). Currently, Sor is the first-line treatment available for stage C patients of Barcelona Clinic Liver Cancer (BCLC) (10). However, the therapeutic potential of Sor is limited to the high cost, low tumor response and the significant side effects (11, 12). Thus, there is a need to find an effective neo-adjuvant to enhance the tumor response and to reduce the side effects of Sor.

Resveratrol (trans 3’,4’,5’-trihydroxystilbene, Res) is a polyphenol compound, which can be found naturally in food and beverage. It is widely recognized as one of the health-promoting components (13, 14). Res has anticancerous activity at multiple stages of tumor development and progression as well as minimal toxicity to normal cells (15, 16). In addition, studies have shown that Res enhances both growth inhibition and cytotoxic activities of several chemotherapeutic agents, including daunorubicin and doxorubicin (DOX), with negligible side effects on normal cells (17, 18). Moreover, it has been shown that Res sensitized the aerobic glycolytic HCC cells to Sor for inducing mitochondria-associated apoptosis through reducing hexokinase 2 (HK2) expression (15).

Recent studies suggest that Res inhibits HCC cell lines’ proliferation through AMP-activated protein kinase (AMPK) activation (19, 20). AMPK plays a central role in cellular energy homeostasis and serves as an essential regulator of metabolic activities in controlling tumor cell growth and proliferation (21, 22). AMPK activation is required for the phosphorylation by protein kinase A (PKA), which has been indicated to regulate many aspects of cell functions, including signal transduction, metabolism, and gene expression (23, 24). The eukaryotic elongation factor 2 kinase (eEF2K) is one of the downstream molecule of AMPK signal pathway and is a CaM-dependent protein kinase III. It phosphorylates and inhibits the function of the eukaryotic elongation factor 2 (EEF2), thus regulating translation and protein synthesis (25, 26). There are papers that have investigated the effects of Sor on AMPK-associated signaling pathways, such as NAD/SIRT1/AMPK axis, AKT and AMPK phosphorylation, TFAM and AMPK, ATP-AMPK-mTOR-SREBP1, and NOD2-AMPK (27–31). However, the combined effects of Res and Sor on the PKA/AMPK/eEF2K signaling pathway during the HCC cell proliferation have not been analyzed to date. Here, we examined whether Res can enhance the inhibitory effect of Sor on HCC cell lines through PKA/AMPK/eEF2K pathway *in vitro* and *in vivo*.

## Materials and methods

### Materials

Res was purchased from LC Laboratories (Woburn, MA, USA). Sor was purchased from Selleck Chemicals (Houston, TX, USA). RPMI 1640 medium and fetal bovine serum (FBS) were purchased from Hyclone (GE Healthcare, USA). Cell Counting Kit-8 (CCK-8) was obtained from Dojin Chemical Co. (Kumamoto, Japan). Annexin V-FITC/propidium iodide (PI) apoptosis assay kit was bought from Roche (Roche, USA). PI and other reagents were purchased from Sigma-Aldrich (Sigma, USA). Antibodies to CDK2, CDC25A, CyclinA, caspase-3, caspase-8, caspase-9, PKA, eEF2K, p-AMPK, and AMPK were purchased from Cell Signaling Technology (Massachusetts, USA).

### Cell culture and treatment

Human HCC cell lines of HepG2 and Huh7, which were authenticated by short tandem repeat (STR) method, were purchased from the cell bank of the Chinese Academy of Science (Shanghai, China). HepG2 and Huh7 were grown in RPMI 1640 medium supplemented with 10% FBS, 100 units/mL penicillin, and 100 mg/mL streptomycin. Cells were incubated in a humidified atmosphere of 5% CO^2^ at 37°C.

Based on the previous published studies (14, 15), for the cell viability assay, HepG2 and Huh7 cell lines were treated with Res and Sor at the concentration of (0, 0.1, 1, 10, 20, 40, 80, and 100 μM) and (0, 2.5, 5, 10, 20, 40, 80, and 100 μM), respectively, for 24, 48, and 72 h. For the combination treatment, 80 μM concertation of Res was selected and combined with 2.5, 5, and 10 μM Sor, and the cells were treated for 48 h.

### Cell viability assay

Cell viability was determined by the CCK-8 method according to the manufacturer’s instructions. Cells were plated and treated with Res and Sor alone, and combination treatment of Res and Sor at different concentrations as described earlier. After incubation, 10 µL of CCK-8 was added to each well and measured absorbance at 450 nm using a Multimode Microplate Reader. Cell viability was calculated from the optical density readings of different treatment groups.

### Cell cycle analysis

After treatment with Res and Sor alone, and in combination treatment for 48 h, HCC cell lines were harvested, washed with PBS, and fixed in 80% ethanol at −20°C overnight. Then, the cells were centrifuged, washed with phosphate buffered saline (PBS), and permeabilized with 0.25% Triton X-100. Furthermore, the cells were incubated with PI (20 μg/mL) supplemented with RNase A (50 μg/mL) for 30 min at room temperature. The relative DNA content was assayed using a BD FACSVerse (BD Biosciences, Heidelberg, Germany) flow cytometer. Cell cycle distribution was analyzed using the FCSExpress 4 Flow Research software.

### Cell apoptosis assay

To determine cellular apoptosis, an Annexin V-FITC apoptosis detection kit with PI was used according to the manufacturer’s instructions. In brief, HCC cells were harvested and washed twice with cold PBS and resuspended in 500 µL binding buffer. Thereafter, the cells were mixed with 5 µL annexin V-FITC and 5 µL PI for 15 min in the dark. The Fluorescence Activating Cell Sorter (FACS) caliber flow cytometer (BDFACS Canto II, BD Biosciences) and modft software were used to analyze the apoptotic cells.

### Western blotting analysis

Regulatory proteins were analyzed by western blotting technique. Western blotting was carried out using the standard protocol. Briefly, cell lysate was prepared in Radio-Immunoprecipitation Assay (RIPA) lysis buffer. Membranes were incubated with primary antibodies overnight at 4°C. Then, the membranes were incubated with horseradish peroxidase-conjugated secondary antibody, probed using enhanced chemiluminescence (ECL) western blotting detection system (Millipore, MA, USA), and visualized using the BioSpectrum AC imaging system.

### In vivo xenograft experiments

Animal experiments were performed according to the protocols approved by the Animal Care and Use Committee of Xi’an Jiaotong University. Female BALB/c (*nu/nu*) mice, 5–6 weeks old, were purchased from Experimental Animal Center of Xi’an Jiaotong University (Shaanxi Province, China). The mice were housed with a light/dark cycle of 12/12 h and allowed free access to rodent chow and water. HepG2 cells were cultured and harvested, washed with PBS, and resuspended in PBS. After anesthetization, 5 × 10^6^/mL cells were injected subcutaneously into the posterior hind flank region of mice. When the tumor masses became established and palpable, the mice were randomly divided into four groups as follows: control (Con, treatment vehicle of 0.9% sodium chloride plus 1% dimethylsulfoxide (DMSO)), Res (20 mg/kg, dissolved in vehicle) alone (22), Sor (25 mg/kg, dissolved in vehicle) alone (32), or combination treatment of Res (intraperitoneal injection) and Sor (oral administration) twice per week for 3 weeks. Tumor volumes and body weights were measured, and the relative volumes and weights were indicated in the experiment. A TUNEL and DAPI staining was used to assay the apoptotic cells in tumor.

### Statistical analysis

Data are expressed as mean ± standard deviation (SD) of at least three independent experiments. We used Student’s *t-*test and Prism 5 software. Differences were considered significant at a *P*-value of <0.05.

## Results

### Antiproliferative activity of Sor and Res in HCC cell lines

**Fig. 1:**
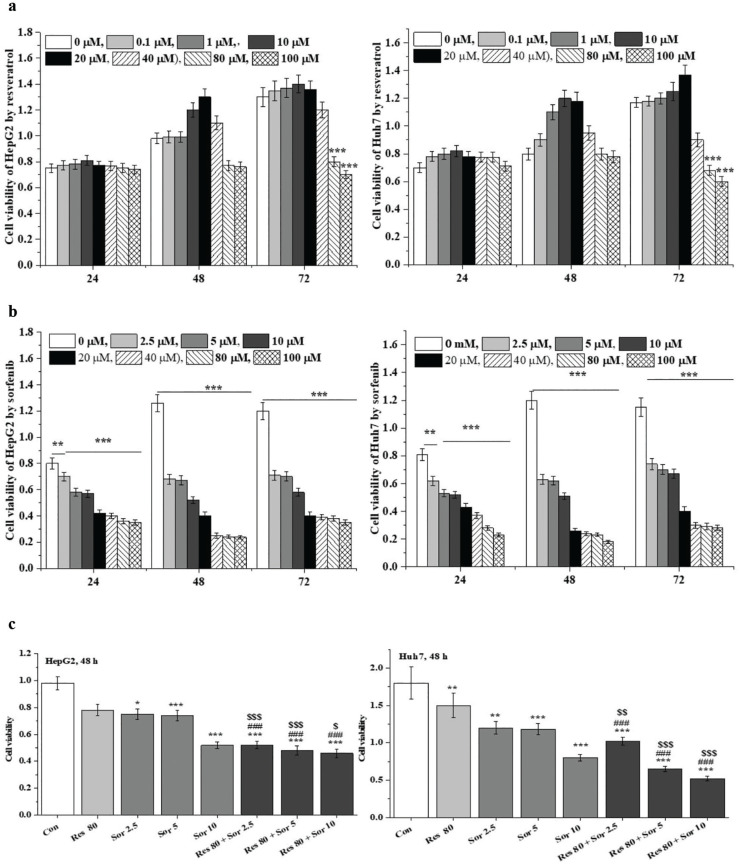
Inhibitory effect of Res, Sor, and Res combination with Sor treatments on proliferation in HepG2 and Huh7 cells. The CCK-8 method was used to assay the cell proliferation, which expressed as cell viability. HepG2 (a) and Huh7 (b) cells were treated at 0–100 µM of Res and Sor alone for 24, 48, 72 h. (c) 80 µM Res combination 2.5–10 µM Sor combination treatment for 48 h in HepG2 and Huh7 cells. Data are expressed as mean ± SD (*n* = 3). ^*^P < 0.05, ^**^P < 0.01, and ^***^P < 0.001 versus the control group. ^###^*P* < 0.001 versus Res treatment group. ^$^*P* < 0.05, ^$$^*P* < 0.01, and ^$$$^P < 0.001 versus the corresponding Sor treatment group.

Cell proliferation was presented as the value of cell viability. The effects of Res and Sor alone on cell viability of HepG2 and Huh7 cell lines are shown in Fig. 1a, b, respectively. The cell viability was significantly decreased (*P* < 0.001) at 80 and 100 μM Res treatment in HepG2 cells for 72 h. Sor treatment significantly decreased (*P* < 0.01 and *P* < 0.001) the cell viability at 2.5–100 μM for 24–72 h in Huh7 cells compared to the control cells (0 μM). Based on the inhibitory effects of Res and Sor treatments alone, the combination treatment of Res (80 μM) and Sor (2.5, 5, and 10 μM) was carried out and assayed for 48 h (Fig. 1c). Significant synergistic antiproliferative effects (*P* < 0.05, *P* < 0.01, and *P* < 0.001, respectively) on the cells were observed in comparison with the corresponding Res and Sor treatments alone.

### Res enhances induction of S phase arrest and reduction of associated regulatory proteins by Sor in HCC cell lines

**Fig. 2:**
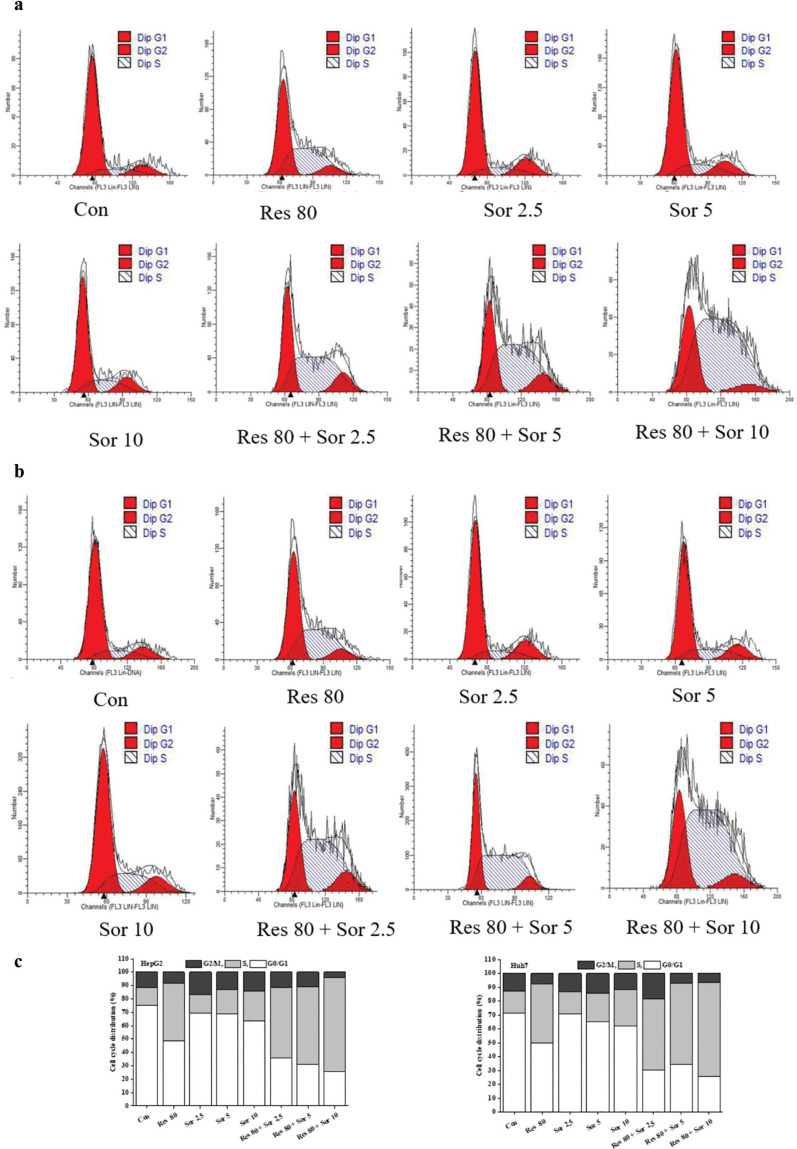
Inhibitory effect of Res, Sor, and Res combination with Sor treatments on cell cycle. (a, b) Cells were harvested, stained with PI, and subjected to flow cytometry at an interval of 6 h, and representative photographs were shown (*n* = 3). (c) The variation mean values of different phases of cell cycle distribution.

Distribution of cells in various phases of cell cycle after treatment with Res at 80 μM, Sor at 2.5, 5, and 10 μM, and Res combined with Sor for 48 h is shown in Fig. 2. Representative photographs (Fig. 2a, b) show notably decreased cells at G0/G1 phase, whereas notably increased cells at S phase in HepG2 and Huh7 cell lines after 80 μM Res treatment (Fig. 2c). Sor treatment slightly decreased cells at G0/G1 phase and increased cells at S phase in both HCC cell lines. Notable decrease at G0/G1 phase and an obvious accumulation of cells at S phase were observed after combined treatment of Res and Sor for 48 h in both cell lines compared to the Res and Sor alone treatments (Fig. 2c).

**Fig. 3:**
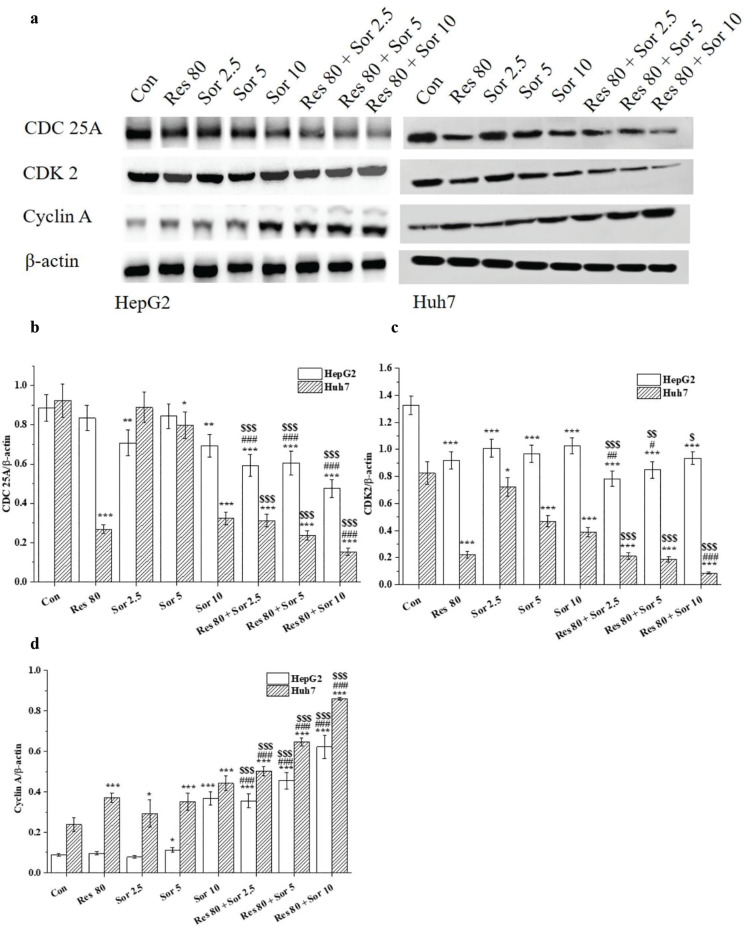
The cell cycle regulatory proteins of CDC 25A, CDK2, and cyclin A in HepG2 and Huh7 cells. (a) The representative photograph of western blotting. (b–d) Analysis of results. Data are expressed as mean ± SD (*n* = 3). ^*^P < 0.05, ^**^P < 0.01, and ^***^P < 0.001 versus the control group. ^#^*P* < 0.05 and ^###^*P* < 0.001 versus Res treatment group. ^$^*P* < 0.05, ^$$^*P* < 0.01, and ^$$$^*P* < 0.001 versus the corresponding Sor treatment group.

Regulatory proteins of CDK2, CDC25A, and cyclin A were shown as in Fig. 3a. Levels of CDK2 and CDC25A proteins were decreased, and for cyclin A, they were increased after Res or Sor treatment alone, and combined treatment in the HCC cell lines (Fig. 3b–d). Significant reductions or increments were also observed in combination treatment for these regulatory proteins (*P* < 0.05 and *P* < 0.001) compared with the Res and Sor alone treatments (Fig. 3b–d). The results showed cell cycle arrest at S phase, and this effect was partly associated with CDK2, CDC25A, and cyclin A proteins.

### Res enhances apoptosis and activate caspase-3, caspase-8, and caspase-9 proteins in Sor-treated HCC cell lines

**Fig. 4:**
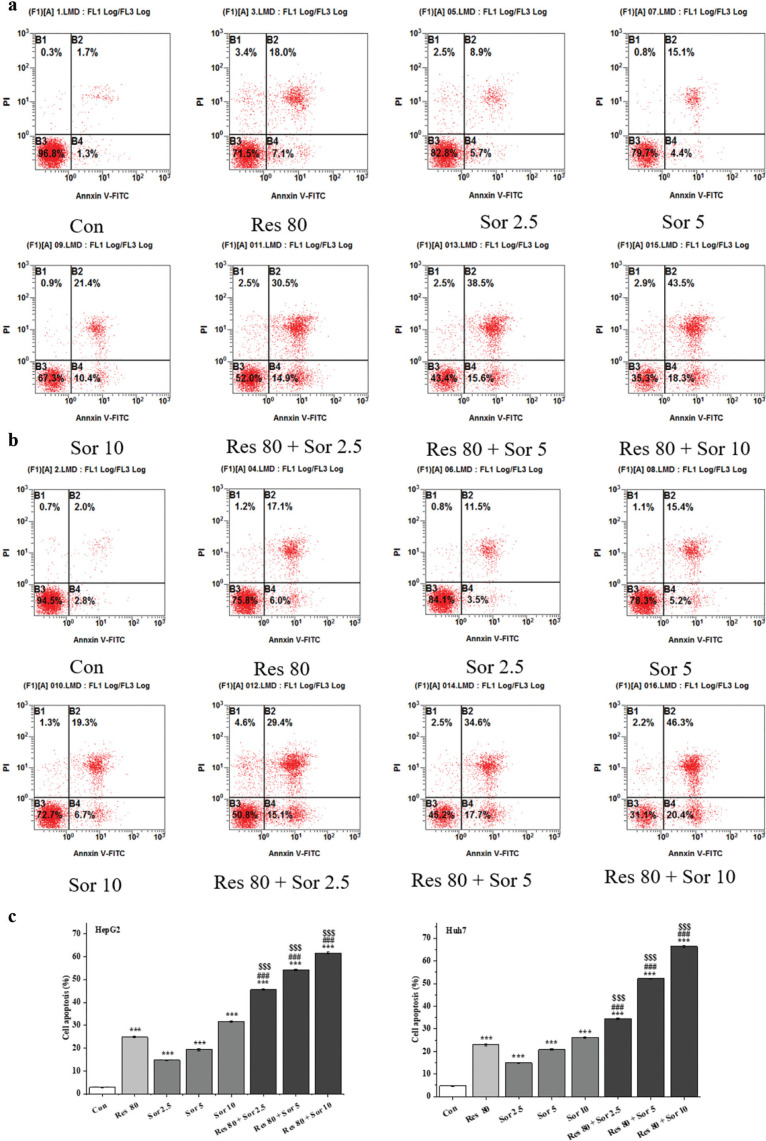
Inhibitory effect of Res, Sor, and Res combination with Sor treatments on apoptosis in HepG2 and Huh7 cells. (a, b) Representative photographs of annexin V-FITC/PI assay to identify apoptotic cells. (c) Analysis of the apoptotic cells (all annexin-V positive cells). Data are expressed as mean ± SD (*n* = 3). ^***^P < 0.001 versus the control group. ^###^*P* < 0.001 versus Res treatment group. ^$$$^*P* < 0.001 versus the corresponding Sor treatment group.

To determine whether Res could enhance the apoptosis induced by Sor, HepG2, and Huh7, cells were treated with Res, Sor, and combination of Res and Sor, then stained with annexin V-FITC and PI, and analyzed by flow cytometry (Fig. 4a, b). The percentage of apoptotic cells (all annexin V-FITC staining cells) was significantly increased (*P* < 0.001, Fig. 4c) in both cell lines after Res, Sor, and combination treatments. Especially, combination treatment group resulted in significant increments (*P* < 0.001) in apoptotic cells compared with Res and Sor alone treatment groups.

**Fig. 5:**
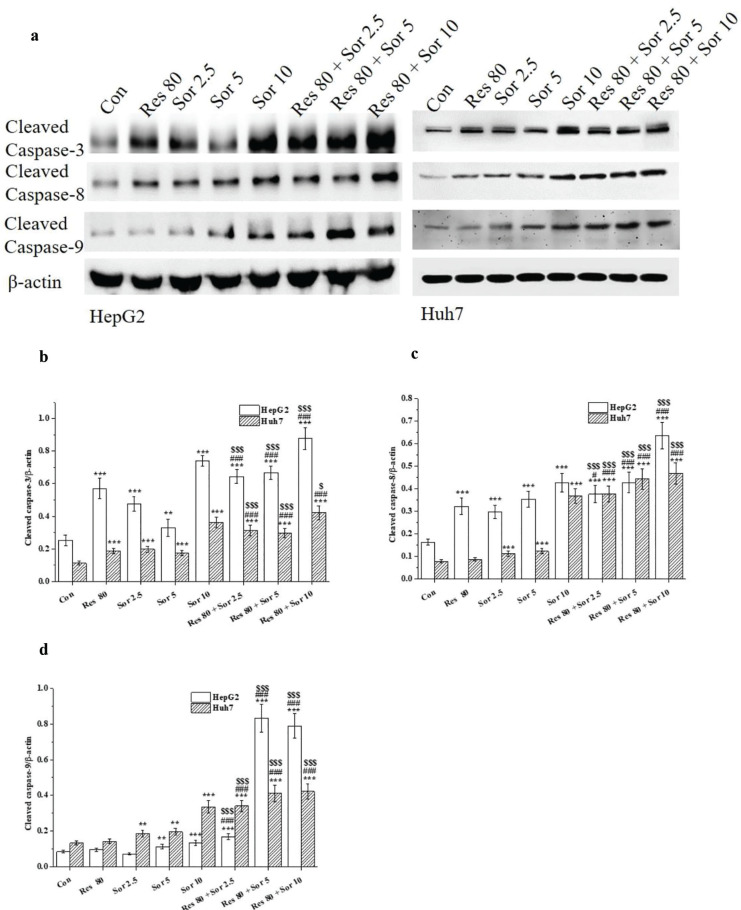
Induction of the apoptotic proteins of cleavage caspase-3, caspase-8, and caspase-9. HepG2 and Huh7 cells were treated with 0 μM (Con.), 80 μM Res, Sor at the concentrations of 2.5, 5, and 10 μM, and Res combination different concentrations of Sor for 48 h. (a) Representative photograph of western blotting. (b–d) Analysis of results. Data are expressed as mean ± SD (*n* = 3). ^**^P < 0.01 and ^***^P < 0.001 versus the control group.^#^*P* < 0.05 and ^###^*P* < 0.001 versus Res treatment group. ^$^*P* < 0.001 and ^$$$^*P* < 0.001 versus the corresponding Sor treatment group.

Cleaved caspase-3, caspase-8, and caspase-9 protein levels (Fig. 5a) were significantly increased in both cell lines after treatment with Res and Sor alone for 48 h (Fig. 5b–d), except cleaved caspase-8 in Huh7 cells treated with Res, cleaved caspase-9 in both HCC cell lines treated with Res, and in HepG2 cells treated with 2.5 μM Sor. The levels of above-mentioned increased proteins were further enhanced significantly (*P* < 0.05 and *P* < 0.001) in the combination treatment of Res and Sor in both cell lines compared with Res and Sor alone treatments.

### Res reduces the expression of PKA, eEF2K, and p-AMPK proteins in Sor-treated HCC cell lines

**Fig. 6:**
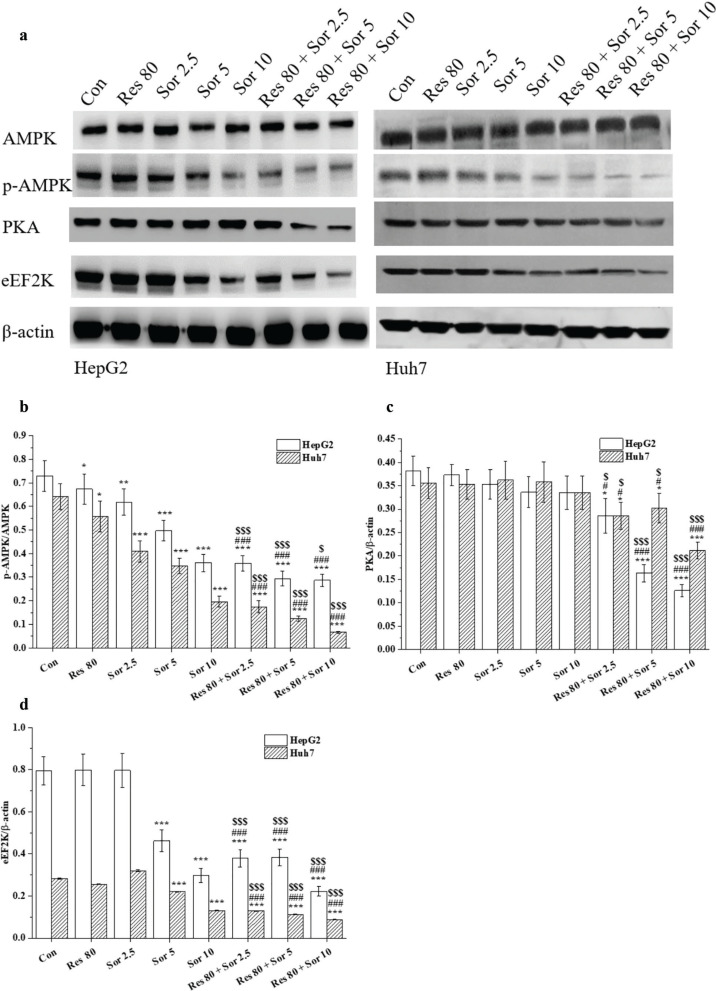
Downregulation effect of Res, Sor, and Res combination with Sor treatment on PKA/AMPK/eEF2K signal in HepG2 and Huh7 cells. Single 80 µM Res, 2.5–10 µM Sor, and combination of Res and Sor treatments for 48 h in HepG2 and Huh7 cells. Western blotting analysis to determine the expression of PKA, p-AMPK/AMPK, and eEF2K proteins. Data are expressed as mean ± SD (*n* = 3). ^*^P < 0.05, ^**^P < 0.01, and ^***^P < 0.001 versus the control group. ^#^*P* < 0.05 and ^###^*P* < 0.001 versus Res treatment group. ^$^*P* < 0.05 and ^$$$^*P* < 0.001 versus the corresponding Sor treatment group.

As shown in Fig. 6, the protein levels of eEF2K were significantly decreased after 5 and 10 μM Sor and combination treatments compared with the untreated cells (Con.) for 48 h. Protein levels of PKA and eEF2K were markedly decreased (*P* < 0.05 and *P* < 0.001) in the combination treatment compared to the Res and Sor alone treatments. The ratio of p-AMPK/AMPK showed a marked reduction (*P* < 0.05, *P* < 0.01, and *P* < 0.001) in single Res and Sor alone treatments, and combination treatment of Res and Sor in both HCC cell lines compared to the untreated cells (Con.), respectively. Similarly, the combined treatment of Res and Sor showed a significant reduction (*P* < 0.05 and *P* < 0.001) in this ratio compared with the Res and Sor alone treatments.

### Res along with Sor synergistically enhances the inhibition of tumor growth in vivo

**Fig. 7:**
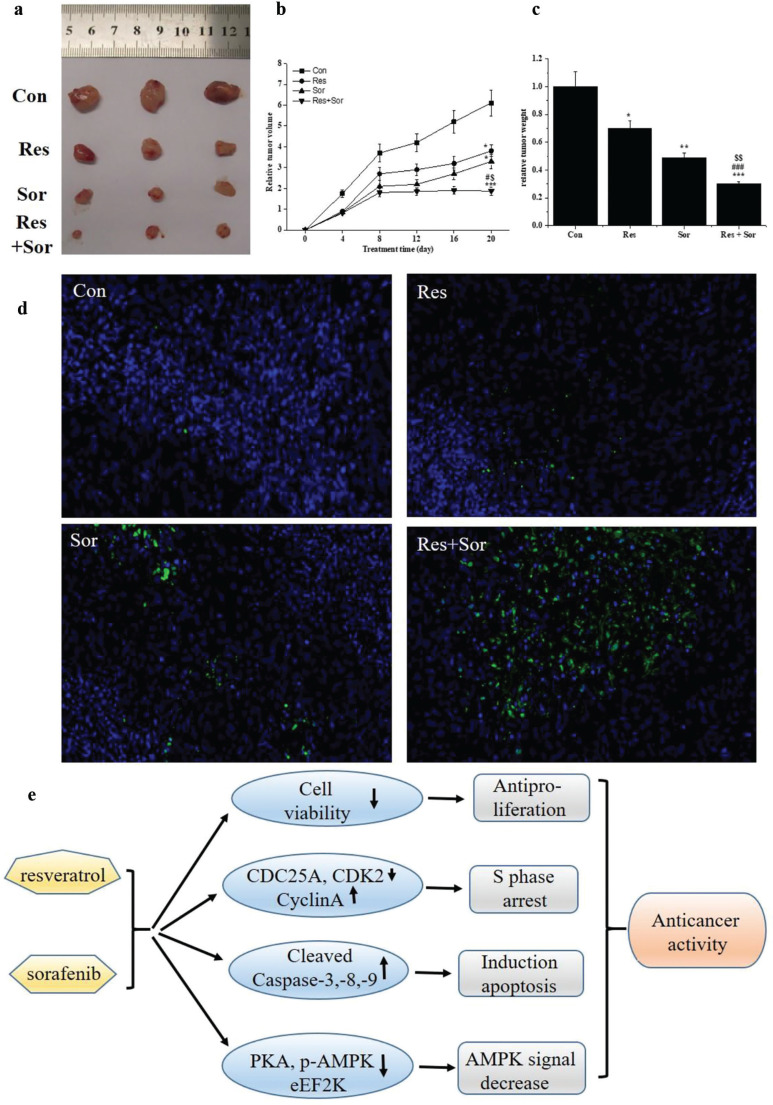
Inhibition of Res, Sor, and Res combination with Sor on HepG2 xenograft growth and on apoptosis in vivo. (a) A representative photograph of a tumor at the end of the experiment. (b, c) The relative tumor volume and tumor weight, respectively. (d) Representative merge photographs of apoptotic cells detected with TUNEL and DAPI staining (200×) in the different groups. (e) Schematic representation of the mechanism of Res combined with Sor on HCC cells. Data are presented as the mean ± SD, *P < 0.05 and ***P < 0.001 versus Con group. ^#^*P* < 0.05 versus Res group. ^$^*P* < 0.05 versus Sor group. (e) Possible mechanism of combination of Res and Sor on HCC cells.

The synergistic effect of Res on Sor to inhibit the tumor growth was evaluated *in vivo*. HepG2 cells were used to generate tumor xenografts in BALB/c nude mice (Fig. 7a). The relative tumor volumes and tumor weights were decreased after Res and Sor alone treatments, and simultaneous combined treatment compared with control group. The relative tumor volumes and tumor weights were significantly decreased (*P* < 0.05) in combination treatment compared to Res and Sor alone treatments (Fig. 7b, c). The apoptotic effect was assayed by TUNEL and DAPI staining. As shown in Fig. 7d, combination treatment notably induced apoptosis in cells compared with Res and Sor alone treatment groups. The findings demonstrate that Res may enhance the ability of Sor to inhibit tumor growth *in vivo*.

Based on the previous findings, the possible mechanism of Res combined with Sor treatment in HCC was demonstrated as Fig. 7e.

## Discussion

The antiproliferative and apoptotic activities of Sor and Res alone have been observed in a wide variety of tumor cell types, including HCC cells. Scanty literature is available about the combined effect of Res and Sor on HCC cell lines and its mechanism. In this study, we evaluated and compared different aspects of HCC cell lines via *in vitro* and *in vivo* experiments using Res and Sor alone treatments, and combination treatment.

We found that 80–100 μM Res treatment induced significant inhibition of cell viability for 72 h. This finding is supported by Dai et al. who demonstrated significant inhibition of cell viability in HCC-LM3, Bel-7402, and Huh-7 cell lines after treatment with Res at 80 μM for 24 h (15). Similarly, Sor, the only approved systemic therapy in HCC patients (1, 5), induced significant inhibition of cell viability in HepG2 and Huh7 cell lines after treatment for 24–72 h. Under combination treatment, significant inhibition of cell viability was observed compared to Res and Sor alone treatments in HePG2 and Huh7 cell lines. This suggests that Res could potentiate the inhibitory effect of Sor on HCC cell proliferation. These findings are consistent with a previous study that indicated significant inhibition of cell viability after 20 μM Res and 5 μM Sor combination treatment for 24 h in HCC-LM3 and Bel-7402 (15). Similarly, significant inhibitory effects on cell viability have been observed for Res and Sor combination treatments in renal carcinoma cell lines of Caki-1 and 786-O (16) and breast cancer cell line of MCF-7 (12).

The antiproliferative effects of these drugs could be due to their action on cell cycle. We observed that there was a significant increase in S phase cells in both cell lines after treatment with 80 μM Res. This has previously been demonstrated that where S phase was arrested after 10 μg/mL Res treatment in Huh7 cell (32). We also observed that the percent of S phase cells was increased, though the percent of G0/G1 cells was decreased in both HCC cell lines after Sor treatment. These findings are in agreement with previous findings about the effect of Sor on cell cycle distribution in HepG2 and Huh7 cell lines (33, 34). However, another study has shown that Sor treatment for 24 h resulted in cell-cycle arrest with a proportional increase in G0–G1 phase and a decrease in S phase in Hep 3B, Hep G2, SK-Hep1, and Huh7 cell lines (35). These findings suggest that the cell cycle alterations are dependent on cell types and treatments (33). Our results also implied that the DNA synthesis was retarded (33). The effect of cell cycle arrest at S phase was very prominent in the combined treatment group, which proved the evidence of Res working synergistically with Sor in cell cycle arrest. Deregulated cell cycles often occur in cancer cells and consequently are a target for cancer drugs (36). Thus, combination treatment of Res with Sor suggests its potential use as anticancerous drugs. Corresponding to the changes of the cell cycle distribution, reduction in CDC25A and CDK2 proteins, thus, suggests the inhibitory effects these drugs on CDK2/cyclin A. These findings are consistent with previous studies where Res treatment caused a reduction in cyclin E and CDK2 expression (32) and Sor treatment reduced levels of CDC25A in HepG2 cell (37). CDC25A plays an important role in cell proliferation by interacting with cyclin A/CDK2 complex to promote S phase cell cycle progression. The decrease in CDK2 activity may be due to the CDC25A degradation, which is likely to contribute to the S-phase checkpoint (38, 39). Therefore, the results of the regulatory proteins may further confirm the variations at G0/G1 and S phases. The findings suggest the CDC25A-cyclin A/CDK2 signaling pathway involvement in S phase arrest after Res and Sor combination treatment in HCC cell lines.

We further investigated Res and Sor alone, and combination treatments on the apoptotic effects. Annexin V/PI double staining assay indicated Res and Sor alone treatments-induced apoptosis in HCC cells, but this phenomenon was much more prominent in the combined treatment groups. Our findings are in line with other studies where Res and Sor alone treatments have been demonstrated to be effective in suppressing HCC cells proliferation and inducing apoptosis (15, 32, 34, 37). Res has also been reported to enhance the effect of Sor on the induction of apoptosis in renal carcinoma cell line of 786-O and in breast cancer cell line of MCF-7 (12, 16). Notably, combination treatment can synergistically increase the cleavage of caspase-3, caspase-8, and caspase-9 compared to Sor and Res alone treatments. The apoptotic caspases, including caspase-3, caspase-8, and caspase-9, play a crucial role in the regulation of programed cell death (40). Our results confirm the synergistic enhancement of apoptosis induced by combined treatment with Res and Sor through the activation of caspase cascade signal as reported in 786-O and MCF-7 cell lines (12, 16).

In our study, we found that Res and Sor alone, and combination treatments decreased the expression of PKA, p-AMPK, and eEF2K, but did not affect AMPK levels. Our results suggest the AMPK signal inactivation through the decrease in the levels of PKA, p-AMPK, and eEF2K. However, significant increment of resveratrol (50 μM) on phospho-AMPK in hepG2 cells (20), no significant effects of resveratrol (5 μM) on H4-II-E cells (19), were observed in published studies. This may due to the concentrations and cells used differently in these studies. Similarly, Sor-induced activation of AMPK through phosphorylation has been demonstrated in most HCC or liver cancer (32–36, 41); there is a study that found a significant decrease after the combination treatment of arsenic trioxide (ATO) and Sor in AMPK activation in comparison to ATO treatment alone (42). This may be due to the status of low AMPK activation in HepG2 and huh7 cell lines that coordinate with other kinases to promote cell survival (43). It is worth noting that Sor alone and combination treatments notably decreased the expression of eEF2K level in our study. The activity of eEF2K was associated with proliferation, migration, and invasion rates. High expression or overexpression of eEF2K has been showed in malignant cancers. Downregulation of expression or silencing of eEF2K has been demonstrated the inhibition of proliferation, migration, and invasion in many cancer cell lines (44, 45). Inhibition of PKA signaling has been demonstrated to prevent both epithelial-mesenchymal transition and invasion of hepatocarcinoma cells (46). Simultaneously, no different effects or induction of PKA phosphorylation by sorafinib (10–25 mM) in Hep3B cells were also found (47). As for downregulation of PKA, this may be associated with cAMP and adenylyl cyclases, which exert tight control cell growth and survival (48). Importantly, combination treatment of Res and Sor induced significant effect on the inhibition of PKA/AMPK/eEF2K signal activation. Thus, our findings reveal that PKA/AMPK/eEF2K signal pathway may involve in the enhancement of Res and Sor combination treatment in HCC cell lines. The exact mechanism underlying the combinatory effect of Res on Sor in HCC cells needs further investigation.

Finally, we found that the combination treatment led to a significant inhibitory effect of tumor growth and tumor volume compared to treatment with either agent alone. So, our finding suggested that the combination of Res with Sor could achieve a greater therapeutic effect in HCC (15).

In summary, the results of the present study revealed that the combination treatment of Res and Sor induced more notable growth inhibition, S phase arrest, and apoptosis in the HCC cell lines than Res and Sor alone treatments. This was accompanied by the downregulation of CDC25A, CDK2, and PKA/AMPK/eEF2K signal and upregulation of cleaved-caspases in HCC cell lines (Fig. 7e). Combination of Res and Sor exhibited more inhibition in tumor growth than Res and Sor alone treatment *in vivo*. Our findings provide a novel mechanism by which Res potentiates Sor to inhibit growth and induce apoptosis in HCC cells. Our findings suggest a combination of Res and Sor therapy, which may be promising for increasing the tumor response of Sor in the future.
